# The Use of Computer-Driven Technologies in the Treatment of Borderline Personality Disorder: A Systematic Review

**DOI:** 10.3390/jcm11133685

**Published:** 2022-06-26

**Authors:** Alexandre Hudon, Caroline Gaudreau-Ménard, Marissa Bouchard-Boivin, Francis Godin, Lionel Cailhol

**Affiliations:** 1 Department of Psychiatry and Addictology, Faculty of Medicine, University of Montreal, Montreal, QC H3T 1J4, Canada; marissa.bouchard-boivin@umontreal.ca (M.B.-B.); francis.godin@umontreal.ca (F.G.); 2 GMF-U Hochelaga-Maisonneuve, Montreal, QC H1V 1K2, Canada; caroline.gaudreau-menard@umontreal.ca; 3 Institut Universitaire en Santé Mentale de Montréal, Montreal, QC H1N 3M5, Canada; lionel.cailhol.med@ssss.gouv.qc.ca

**Keywords:** borderline, personality disorder, computer-driven, systematic review, treatment

## Abstract

The objective of this study was to perform a systematic review of the effectiveness of computer-driven technologies for treatment of patients suffering from BPD. A systematic literature review was conducted using the Pubmed, EMBASE, PsycNET (PsycINFO), CINAHL and Google Scholar electronic databases for the period from their inception dates until 2022. Thirty studies were selected for abstract screening. Seven studies were excluded for not meeting inclusion criteria. The remaining 23 studies were fully assessed, and 12 were excluded. Therefore, 11 studies were included in the analysis of the effectiveness of computer-driven technologies, which encompassed mobile applications, telehealth interventions, internet-based interventions, virtual reality MBT and dialogue-based integrated interventions. Computer-driven interventions are showing signs of effectiveness in the treatment of BPD symptoms. The limited number of articles found on the subject demonstrates a need for further exploration of this subject.

## 1. Introduction

Borderline personality disorder (BPD) is a widespread mental health disorder with a prevalence ranging from 0.5 to 1.4% in the general population [1]. In the psychiatric population, its prevalence is much greater, ranging from 15 to 28% [2]. This disorder is hypothesized to be developmental and is characterized by impulsive behaviours, auto-mutilation and suicidal tendencies [3,4]. BPD is characterized mainly by patterns of unstable relationships, fear of abandonment, rapid changes in self-identity, impulsive and risky behaviour, wide mood swings and ongoing feelings of emptiness [4]. During emotional dysregulations, patients with BDP can impulsively perform non-planned suicidal gestures. Several studies suggest that the prevalence of completed suicide in patients diagnosed with BDP is around 10% [5,6]. In patients with psychiatric comorbidities, the prevalence of suicide attempts can be much higher. For example, a recent comparative study highlighted that the lifetime prevalence of suicide attempts can be up to 90% for patients with concurrent bipolar disorder and BPD [7]. Patients suffering from BDP also present a heavy impact on healthcare systems, family units and friendships [7]. A register-based cohort study of 2756 patients suffering from BPD reported that the societal cost is around 16,728 euros per patient per year, and this number can reach up to 28,026 euros [8,9].

Many guidelines are readily available on the treatment of patients diagnosed with BDP. However, in the ways that the suggested guidelines can be applied to help these patients are unclear [9]. Psychotherapy is the key component in the treatment of BPD [10]. Different types of psychotherapy have been found to be effective to ease impulsivity and emotional dysregulation (BDP severity) [11]. Dialectic behavioural therapy (DBT), invented by Marsha Linehan in the late 1980s, is the most highly recommended therapy for these patients is focused around four major aspects: distress tolerance, emotional regulation, mindfulness and interpersonal effectiveness [12]. Mentalization-based therapy (MBT) has also been found effective in reducing psychiatric symptoms in patients with BDP [13]. Currently, no psychopharmaceutical treatments are recommended for this disorder [14]. However, second-generation antipsychotics (e.g., quetiapine, olanzapine) and serotoninergic antidepressants (e.g., citalopram, sertraline) have been found to have uses in reducing core symptoms of BPD [15]. Due to the complexity of BPD, many patients end up medicated without any significant improvements [16,17]. Despite the wide array of psychotherapy and medication available, some patients remain treatment-resistant and need further innovative approaches in light of the fact that BPD can lead to serious life-threatening consequences. Treatment resistance has been challenged by several investigators, who have highlighted the multiplicity of individual differences, the various treatment approaches available and the lack of consideration given to specific patients’ characteristics.

Computer-driven technologies are used in different areas of medicine to personalize treatments and address accessibility and applicability problems [18]. In psychiatry, for instance, several modalities are already used to help clinicians in the diagnosis and treatment of psychopathologies. Several computer-driven technologies are also used in the field of psychotherapy. For example, novel therapies, such as virtual reality therapies, are used in the treatment of anxiety disorder, schizophrenia and phobias [19,20,21]. For accessibility, modalities such as internet-based cognitive behavioural therapy are emerging [22]. Mobile solutions are also available for anxiety and depressive disorders [23]. A scoping review of technology-based psychosocial interventions for patients with BDP was undertaken in 2020 and identified potential software packages in terms of their research development level, with limited evidence provided about the effectiveness of these interventions [24]. Computer-driven technologies are evolving fast and there is a need to explore the effectiveness of these interventions. To our knowledge, there are no studies that examine the effects of computer-driven modalities on BPD symptoms. The effects of these technologies could be potentially helpful in the treatment of patients with complex disorders like BPD.

The objective of this study was to perform a systematic review of the effectiveness of computer-driven technologies for medical and psychological treatments of patients suffering from BPD. This would provide a broad overview of novel approaches for the treatment of BPD and could provide hints for future explorations of personalized medicine for patients diagnosed with this disorder. 

## 2. Materials and Methods

### 2.1. Search Strategies

A systematic literature review was conducted using the Pubmed, EMBASE, PsycNET (PsycINFO), CINAHL and Google Scholar electronic databases for the period from their inception dates until 2022. The Preferred Reporting Items for Systematic Reviews and Meta-Analyses (PRISMA) statement was used to guide the study design. Indexing (MeSH) terms and text words were used in the inquiries with the electronic databases for the key concepts of BPD (e.g., borderline, personality, BPD) and computer-driven technologies (e.g., internet-based interventions, computers, mobile, cell phone, telemedicine, virtual reality, information technology). These conceptual entities were selected as they best encompass the modalities for computer-driven interventions in the treatment of BDP. Further studies were identified via cross-referencing. A complete research strategy can be found in Appendix A. The search methodology was developed by AH and a librarian specialized in psychiatry at the Institut universitaire en santé mentale de Montréal. Searches were completed by AH and cross-validated by CGM in March 2022. If there was a disagreement over the inclusion or exclusion of a study, a third-party reviewer was brought in. No geographical restriction was applied. Only articles written in French or English were selected. The review was not registered.

### 2.2. Study Eligibility

The following criteria were used for the inclusion of studies in this review: (1) the study involved a computer-driven intervention for the treatment of patients suffering from BPD; (2) the study was conducted with the intent of addressing at least one symptom of BPD or general suffering from BPD; (3) the study included a qualitative or quantitative tool for the assessment of the effectiveness of an intervention on the symptoms of BPD; (4) the study was an original article; (5) the study included an engaging or immersive component involving the patient. Studies that used several different types of computer-driven technologies were included. Unpublished studies were excluded.

### 2.3. Data Extraction

Data were extracted with the help of a standardized Excel form and cross-validated by AH and CGM. The type of intervention, number of participants, outcome of interest, indicators of interest, effect size (if applicable) and general conclusion were collected. 

### 2.4. Quality Analysis of Studies

The methodological quality of the non-randomized and randomized controlled studies identified was analysed based on an adaptation of the Cochrane Collaboration Tool for Assessing the Risk of Bias [25]. AH extracted study characteristics and CGM assessed risk of bias.

## 3. Results

### 3.1. Description of Studies

**Figure 1:**
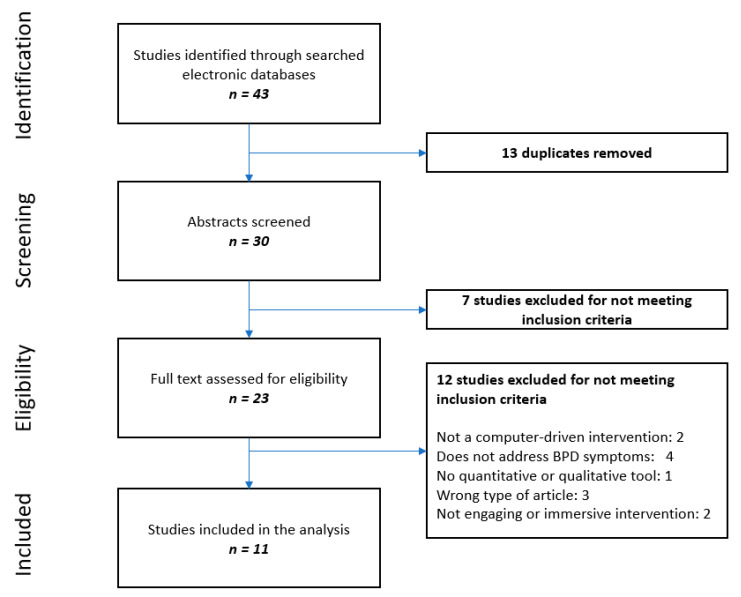
Flowchart of the study selection process.

The systematic review assessed studies that evaluated the effectiveness of computer-driven interventions on BPD symptoms. After the removal of duplicates, 30 studies were selected for abstract screening. This process eliminated seven studies, which were excluded for not meeting inclusion criteria, as they did not include a computer-driven intervention. The remaining 23 studies were fully assessed, and 12 studies were excluded (two studies did not assess a computer-driven intervention, four did not address BPD symptoms, one did not assess the effectiveness of the intervention, three were protocols and two did not involve engaging (or immersive) interventions).Therefore, 11 studies were included in the analysis. A flowchart for the inclusion of studies in this review is displayed in Figure 1. This process highlighted the small number of studies on this topic and its emerging nature. The complete details for the studies that were included are presented in Table 1. Most of the studies addressed the effectiveness of mobile applications. Two studies evaluated internet-based interventions for self-management of BPD symptoms and psychoeducation. One study evaluated telehealth interventions for treatment of patients in a partial hospital setting. A single study offered an overview of a virtual reality-based MBT with the use of an avatar. Finally, two studies addressed the effectiveness of a dialogue-based integrated intervention that uses the concept of schema therapy.

**Figure 2:**
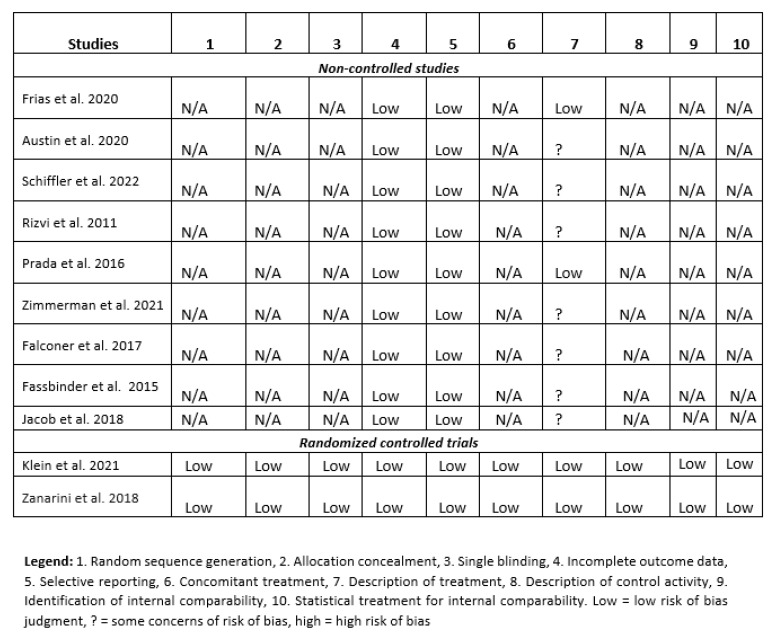
Methodological analysis of the identified studies [26,27,28,29,30,31,32,33,34,35,36].

Figure 2 summarizes the different domains relating to the methodological qualities of the identified studies. The methodological aspects of the two randomized clinical trials were covered adequatly. Most of the methodological aspects of the non-randomized studies were also well-covered, despite widely varying design methodologies. However, for seven of the eight non-randomized studies, the assigned intervention was superficially described.

### 3.2. Computer-Driven Interventions

#### 3.2.1. Mobile Applications

Five mobile applications were identified in this systematic review. 

The first mobile application assessed was the Rapid Intervention Guidelines using Health Technology for Borderline Personality (B-RIGHT) developed by Frias et al., which is a mobile application for self-management of emotional crises [26]. This mobile application, based on cognitive-behavioural techniques, was tested in a pilot study by 25 outpatients with diagnoses of BPD. As well as the usability, they evaluated BPD symptoms (clinical features) using the Borderline Symptom List (BSL), the Difficulties in Emotion Regulation Scale and Beck’s Depression Inventory (BDI). The application demonstrated promising results for emotion regulation and improvement of depressive symptoms. 

Three mobile applications consisted of DBT integrations. Austin et al. assessed the overall experience and facilitation of therapeutic alliance (integration of DBT skills that are relevant to patients with BPD symptoms) among 24 outpatients diagnosed with BPD receiving DBT via a mobile application [27]. This mixed-method study, using in-depth interviews and questionnaires, revealed that such applications may make many of the therapeutic techniques in DBT treatment easier. Similarly, Schiffler et al. designed a study that included 13 transitional age youth (18–23 year old patients) diagnosed with BPD and assessed their experiences and associated emotions before and after a testing period of 30 days while using a mobile DBT application [28]. However, they highlighted that this mobile application did not bring about any changes in the subjective view of suicidality. Rizvi et al. analyzed changes in emotion intensity and urges to use substances in 22 patients diagnosed with BPD with concomitant substance use [29]. Using the BDI, the Behavioural Confidence Questionnaire and the Brief Symptom Inventory, they demonstrated reductions in emotional intensity from pre-coaching to post-coaching, as well as reductions in urges to use substances, for these patients.

The last mobile application identified was EMOTEO. This mobile application was designed to monitor and reduce aversive tension in patients with BPD by suggesting targeted mindfulness-based exercises. Prada et al. designed a pilot study to assess the aversive tension of 16 patients suffering from BPD via a self-reported questionnaire [30]. The authors reported that this application was user-friendly and efficient in reducing aversive tension in BPD patients.

#### 3.2.2. Internet-Based Interventions

Two studies reported the effectiveness of internet-based interventions for BPD symptoms in patients suffering from BPD.

The first study, by Klein et al., reported a randomized controlled trial study that assessed the effectiveness and safety of internet-based self-management interventions [31]. A group of 204 patients, randomized in two groups (one group receiving the internet-based self-management interventions and the other group receiving care as usual), was assessed using the Borderline Personality Disorder Severity Index (BPDSI) scale pre- and post-intervention. These interventions consisted of self-guided or guided interventions based on evidence-based psychotherapy. No significant differences between the intervention and care as usual were found.

Zanarini et al. assessed the reduction in BPD symptoms in a randomized trial consisting of 80 women with BPD [32]. A group of 40 women received a web-based psychoeducation intervention while the other group received care as usual. The change in BPD symptoms was assessed with the Zanarini Rating Scale for Borderline Personality Disorder. The group who received the web-based intervention reported a significantly greater decline in symptoms in all five studied areas of borderline psychopathology: affective symptoms, cognitive symptoms, impulsivity, interpersonal difficulties and overall BPD symptoms. The authors concluded by stating that internet-based psychoeducation is an effective form of early treatment for reducing the symptom severity of BPD.

#### 3.2.3. Telehealth Intervention

A comparative study designed by Zimmerman et al., conducted as part of the Rhode Island Hospital Adult Partial Hospital Program, compared the use of telehealth formats of ACT and evidence-based psychotherapy techniques, such as CBT and DBT, to their in-person counterparts [33]. This study analyzed the change in BPD symptoms for two groups: 54 in-person patients with BDP and 28 patients with BDP using the telehealth modality. They used the Remission from Depression Questionnaire as the primary outcome. The effect sizes reported for each of the following components were significant: overall symptoms, depression, anxiety, anger, physical pain, positive mental health, functioning, coping skills and well-being. This study demonstrated a significantly greater improvement in functioning in the virtual program group and less improvement in anger.

#### 3.2.4. Virtual Reality MBT

A single study on this topic was identified, designed by Falconer et al. [34]. It consisted of an adjunctive avatar therapy for MBT (avatar-MBT) treating BPD disorder. Avatar-MBT helps the user create a virtual visual representation of their internal and external world with interactive elements. While this study was a mixed-method feasibility study, it outlined several elements pertinent to BPD symptoms by using the 21-item Depression, Anxiety and Stress Scales and a qualitative component. For the 11 outpatients diagnosed with BPD who received this therapy, the statistical analysis revealed no significant change for any self-report measures. However, the qualitative component showed that the participants reported that avatar-MBT had more impact and was more powerful than standard MBT. The authors concluded by stating that avatar-MBT is a promising enhancement for therapy for BPD.

#### 3.2.5. Dialogue-Based Integrated Interventions

In 2015, Fassbinder et al. designed a first case study on Priovi, which is an e-health, dialogue-based, integrated tool that uses the concepts of schema therapy [35]. In this case study, consisting of a female patient with a diagnosis of BPD, BPD symptoms were assessed using the BPDSI. The patient experienced a decrease of 27 points, thus helping the authors to conclude that Priovi could potentially increase treatment intensity and enhance treatment effects. This approach was further studied in 2018 by Jacob et al. [36]. An observational study was realized with 13 participants diagnosed with BPD (11 females and 2 males) and their BPD symptoms were assessed using the BPDSI and BPD-CL scales. A reduction of 9.6 points in the BPDSI on average was noted (Cohen’s D = 1.0) and a reduction of 29.9 in BPD-CL was observed (Cohen’s D = 1.2), demonstrating that Priovi could be a potentially helpful tool in the improvement of BPD symptoms.

### 3.3. Outcomes

Among the 11 identified studies, various computer-driven technologies were assessed. There is still a lack of evidence regarding effectiveness for all the above modalities. The wide range of methodologies used to assess the effectiveness does not permit comparisons between the modalities. There is a greater body of evidence for mobile applications and internet-based interventions as compared to emerging interventions, such as telehealth, virtual reality MBT and dialogue-based integrated interventions. Meta-analysis could not be applied for this review as the identified studies were clinically diverse and because there was a mix of comparisons of a variety of treatments using different tools to assess BPD symptoms.

## 4. Discussion

### 4.1. About the Findings

In this systematic review study, we analyzed 11 studies that assessed the effectiveness of computer-driven technologies, such as mobile applications, telehealth interventions, internet-based interventions, virtual reality MBT and dialogue-based integrated interventions.

Amongst the five reported mobile applications, four showed promising results regarding the improvement of BPD symptoms. However, it is difficult to state their effectiveness due to the numerous limitations in the identified studies, such as sample sizes, qualitative assessments and the various methodologies employed to assess BPD symptoms. The conclusions of these studies are consistent with a systematic literature review on the subject that assessed 16 mobile health applications for various mental health disorders [37]. For patients with BPD symptoms, Frias et al. raised the hypothesis that mobile applications may work as transitional objects [26]. This follows the literature on the subject by highlighting how these applications permit psychological growth despite the absence of relationships, which can occur in patients with BPD experiencing interpersonal conflicts [38]. In the identified studies, emotion dysregulation (or intensity) was reported as the symptom that improved the most for those who used mobile applications. A study examining the use of mobile applications to regulate negative emotions in 287 young adults demonstrated that mobile applications can provide psychological benefits and effective remediation of negative emotions [39]. Such effects, combined with DBT skills that have been proven efficient in reducing BPD symptoms, could be beneficial.

Internet-based interventions revolved around psychoeducation. While Zanarini et al.’s study reported a significant decline in BPD symptoms, the comparative study designed by Klein et al. did not report such improvements for their intention-to-treat protocol [31,32]. However, in Klein et al.’s study, the per-protocol approach demonstrated a significant decline in BPD symptoms, therefore hinting that, if patients are better supported in using the program, effectiveness may be more convincing. Psychoeducation in patients with BPD has been proven to be helpful in a regular care setting, which might explain why the former study did not find any significant changes [40]. However, as both studies suggested, accessibility is often limited, and an internet-based intervention could be interesting for patients with limited access to care providers. This follows the recommendations that interventions for patients suffering from BPD should be made more accessible [41]. This is consistent with a recent scoping review designed by van der Boom and al. (2022), limited to internet-delivered interventions for personality disorders, which highlighted that about 45% of the included studies (N = 11) reported changes in symptoms [42].

Telehealth interventions have been gaining popularity, especially during the COVID-19 pandemic [43]. For BPD symptoms, only one study addressed telehealth interventions with patients suffering from BPD. The elements of the Remission from Depression Questionnaire were found to have significantly improved in the virtual program that was analyzed by Zimmerman et al. [33]. While it is difficult to explain exactly why the virtual program would yield greater improvements, several studies about telehealth interventions are consistent and show comparable outcomes. For example, a randomized noninferiority trial that compared teleconferencing versus in-person acceptance and commitment therapy for 128 veterans with chronic pain revealed that this noninferiority hypothesis was supported [44]. For BPD patients, it can be hypothesized that the virtual aspect of this modality is less intimidating and, therefore, enables a greater therapeutic alliance, which helps with the reduction of BPD symptoms.

Avatar-based MBT is still a novel avenue for the treatment of BPD symptoms. The major elements of MBT are used in this approach, which is why this format is hypothesized to be effective. The visual aspects of avatar-based MBT might help the patients in the process of mentalization thanks to its immersive component. BPD patients are reported to have difficulties mentalizing [45]. The major qualities of virtual environments are their ecological validity, presenting highly controlled environments, and the enhancement of personalization and engagement [46]. Therefore, these qualities combined with classical MBT, which has already been found to be useful for BPD symptoms, support the observations of Falconer et al. This avenue remains novel and further studies should be conducted on the subject.

With regard to the dialogue-based integrated tool Priovi, the authors reported potential avenues for BPD symptoms. Priovi is based on Schema Therapy. The literature currently supports the effectiveness of Schema Therapy, ranging from assessments of it as a promising avenue to demonstrations of its effectiveness for the reduction of BPD symptoms [47,48,49]. This could explain why the implementation of Priovi was also found promising for the symptoms studied here. However, the accessibility of this type of therapy remains a major difficulty for some patients. Therefore, such a tool could provide an avenue where limited access to therapy is a constraint. Further research should be conducted to compare this implementation to in-person MBT to assess its effectiveness. 

### 4.2. Limitations

This systematic review focused solely on the literature addressing the effectiveness of computer-driven interventions without limitations on the methodologies used for their assessments. Therefore, it was hard to establish comparisons between the identified modalities. The small number of studies identified reflects the novelty of this domain, which should expand with time. The search strategy was limited to medical databases to reflect the main objective. An approach targeting a wider range of practitioners could identify further interventions. 

## 5. Conclusions

Computer-driven interventions are showing signs of effectiveness in the treatment of BPD symptoms. This could yield potential novel avenues in the treatment of patients suffering from BPD. Using computer-driven interventions could be beneficial where accessibility to treatment as usual is not accessible or delayed, and this should be further studied. The promising effectiveness of these interventions demonstrates that they could also be employed as adjuncts to standard therapies and treatment plans. The limited number of articles found on the subject demonstrates a need for further exploration of this subject. Further studies, especially randomized controlled trials, should be conducted to better assess the effectiveness of these novel treatments for BPD symptoms.

## Figures and Tables

**Table 1:** Details for the studies included.

Studies	Intervention Type	Number of Participants	Outcome of Interest	Measurement Indicators	Effect Size	General Conclusion
Frias et al., 2020 [26]	B-RIGHT: mobile app for selfmanaging emotional crises	25 outpatients, 84% female	Satisfaction, usability, emotion dysregulation and depression severity. BSL-23, Difficulties in Emotion Regulation Scale, Beck Depression Inventory	Pearson’s correlation (r) or Spearman’s correlation (rho)	Emotion dysregulation (r = 0.51); depression severity (rho = −0.47)	Promising results. Mobile application may work as a transitional object
Austin et al., 2020 [27]	Mobile app integration into dialectical Behaviour therapy	24 outpatients	Overall experience, facilitate therapeutic alliance	Qualitative	Not applicable	May make many of the therapeutic techniques in DBT treatment easier
Schiffler et al., 2022 [28]	Mobile DBT application	13 transitional age youth (18–23 years old)	Experiences and associated emotions before and after a testing period of 30 days	Qualitative	Not applicable	Did not bring about any changes in the subjective view of suicidality
Rizvi et al., 2011 [29]	Mobile phone application: DBT coach	22 patients with BPD and substance use	Emotion intensity and urges to use substances	Beck Depression Inventory, Behavorial Confidence Questionnaire, Brief Symptom Inventory	Reduction in emotional intensity from precoaching to postcoaching was significant, B = −1.26, SE = 0.20, t(21) = −6.17, *p* < 0.001. Reduction in urges to use substances was also significant, B = −0.92, SE = 0.22, t(21) = −4.22, *p* < 0.001	May be a useful tool for reducing urges to use substances and engage in other maladaptive behaviour
Prada et al., 2016 [30]	EMOTEO: A smartphone application for monitoring and reducing aversive tension in BPD	16 patients with BPD	Aversive tension	Self-reported homemade questionnaire	No effect size reported. Regarding aversive tension, users reported a mean of 4.5 (on a scale ranging from 1 to 5, where 5 is the best outcome) with an SD of 0.71	EMOTEO was user-friendly and efficient in reducing aversive tension in BPD patients
Klein et al., 2021 [31]	Internet-based self-management intervention for BPD	204 patients with BPD, randomized in two groups: care as usual and care as usual + internet-based system	Effectiveness and safety	BPDSI	At 12 months, Cohen’s D of 1.38 for intervention group and 1.02 for the control group	No significant difference between the intervention and care as usual for the intention-to-treat protocol; however there were significant differences in the per-protocol
Zanarini et al., 2018 [32]	Web-based psychoeducation	80 women with BDP, randomized in two groups of 40: with web-based psychoeducation and without web-based psychoeducation	BPD symptoms	Zanarini Rating Scale for Borderline Personality Disorder	Treatment group reported a significantly greater decline in all five studied areas of borderline psychopathology: affective symptoms (z = −2.31, *p* = 0.021), cognitive symptoms (z = −3.20, *p* = 0.001), impulsivity (z = −2.44, *p* = 0.015), interpersonal difficulties (z = −2.15, *p* = 0.032), and overall borderline personality disorder symptoms (z = −2.11, *p* = 0.035)	Internet-based psychoeducation is an effective form of early treatment for reducing the symptom severity of BPD
Zimmerman et al., 2021 [33]	Telehealth treatment of patients with borderline personality disorder in a partial hospital setting: comparative study	54 in-person patients with BDP, 28 patients with BDP using the telehealth modality	Symptoms, depression, anxiety, anger, physical pain, positive mental health, functioning, coping skills, well-being	Remission from Depression Questionnaire	Effect sizes (Cohen’s D) of 1.81, 1.98, 1.48, 1.03, 0.53, 1.45, 1.63, 1.61 and 1.78, respectively	Significantly greater improvement in functioning in the virtual program, less improvement in anger
Falconer et al., 2017 [34]	Avatar-based MBT	11 outpatients	Perspective taking, expression, emotional distancing; 21-item Depression, Anxiety and Stress Scales; Mentalization Questionnaire; semi-structured interviews	Mean and standard deviation analysis via ANOVA; thematic annotations	No effect from the ANOVA. May enhance the therapeutic efficacy of standard MBT	Avatar-MBT is a promising enhancement of therapy for BPD
Fassbinder et al., 2015 [35]	Priovi: dialogue-based integrated structure	1 case example	Skills and experiences in patients with BPD	Borderline Personality Disorder Severity Index (BPDSI); WHODAS 2.0, overall score; Schema Mode Inventory	Decrease of 27 points in the BPDSI	Could potentially increase treatment intensity and enhance treatment effects
Jacob et al., 2018 [36]	Priovi: observational study	13 participants diagnosed with BDP: 11 females, 2 males	BPD symptoms	BPDSI-IV, BPD-CL, qualitative patient interviews, qualitative therapist interviews	Reduction of 9.6 points in the BPDSI (Cohen’s D = 1.0) and of 29.9 in BPD-CL (Cohen’s D = 1.2)	Priovi could be a potentially helpful tool in the improvements of BPD symptoms

## Data Availability

Not applicable.

## Appendix A

**Search strategies**

Concept 1

“Borderline Personality Disorder”[Mesh] OR (Borderline AND (personality[TIAB] OR personalities[TIAB] OR state[TIAB])) OR BPD[TIAB]

Borderline AND (personalit* OR state) OR BPD

Concept 2

“Internet-Based Intervention”[Mesh] OR “Mobile Applications”[Mesh] OR “Cell Phone”[Mesh] OR “Computers, Handheld”[Mesh] OR “Telemedicine”[Mesh] OR “Internet-based”[TIAB] OR “Virtual Reality” [TIAB] OR “VR”[TIAB] OR “Information technology”[TIAB] OR “Computer-based”[TIAB] OR “Technology-based”[TIAB] OR “Web-based”[TIAB] OR Telemedicine[TIAB] OR Tele-medicine[TIAB] OR Telepsychiatry[TIAB] OR Tele-psychiatry[TIAB] OR Ehealth[TIAB] OR e-mental[TIAB] OR Emental[TIAB] OR Mhealth[TIAB] OR ((Online[TIAB] OR “On-line”[TIAB] OR Mobile[TIAB] OR “Cell phone”[TIAB] OR “cell phones”[TIAB] OR Smartphone[TIAB] OR smartphones[TIAB] OR digital[TIAB]) AND (Application[TIAB] OR applications[TIAB] OR App[TIAB] OR apps[TIAB] OR Intervention[TIAB] OR interventions[TIAB] OR Treatment[TIAB] OR treatments[TIAB] OR Therapy[TIAB] OR therapies[TIAB] OR Psychotherapy[TIAB] OR psychotherapies[TIAB] OR Psychoeducation[TIAB] OR psychoeducational[TIAB] OR Psycho-education[TIAB] OR psycho-educational[TIAB] OR care[TIAB] OR service[TIAB] OR services[TIAB] OR clinical[TIAB]))

“Internet-based” OR “Computer-based” OR “Virtual Reality” OR “VR” OR “Information technology” OR “Technology-based” OR “Web-based” OR Telemedicine OR Tele-medicine OR Telepsychiatry OR Tele-psychiatry OR Ehealth OR e-mental OR Emental OR Mhealth OR ((Online OR “On-line” OR Mobile OR “Cell phone*” OR Smartphone* OR digital) ADJ3 (Application* OR App OR apps OR Intervention* OR Treatment* OR Therap* OR Psychotherap* OR Psychoeducation* OR Psycho-education* OR care OR service*OR clinical))
